# Rat embryonic stem cell-based *in vitro* testing platform for mammalian embryo toxicology at pre- and post-implantation stages

**DOI:** 10.3389/ftox.2025.1561386

**Published:** 2025-05-08

**Authors:** Corinne Quadalti, Marzia Moretti, Fabio Ferrazzi, Laura Calzà, Luciana Giardino, Vito Antonio Baldassarro

**Affiliations:** ^1^ Department of Pharmacy and Biotechnology (FaBiT), University of Bologna, Bologna, Italy; ^2^ Interdepartmental Centre for Industrial Research in Health Sciences and Technology ICIR-HST, University of Bologna, Bologna, Italy; ^3^ Department of Veterinary Medical Sciences (DIMEVET), University of Bologna, Bologna, Italy; ^4^ IRET Foundation, Bologna, Italy

**Keywords:** rat embryonic stem cells, developmental toxicity, TBBPA, PFOA, retinoic acid, i*n vitro* models, alternative methods

## Abstract

**Introduction:**

The international guidelines outlining the mandatory developmental toxicology studies of new molecules on pre-implantation, post-implantation and organogenesis phases, require a minimum of 60 pregnant female rats for each molecule to be tested. To date, available in vitro methods still have many limitations, resulting in poor translational power.

**Methods:**

In the present study, an innovative in vitro platform is proposed, based on rat embryonic stem cells (RESCs), which is easy to use and suitable for wide-scale screening, mimicking two different developmental stages: i) pre-implant model (undifferentiated pluripotent cells), ii) post-implant model (neuroectodermal lineage differentiation).

**Results:**

The in vitro platform was validated by testing the toxicity on the pre-implant model of RA itself, as a known teratogen, a member of the environmental pollutant family per- and polyfluoroalkyl substances (PFAS), the perfluorooctanic acid (PFOA), and the endocrine disruptor chemical 2,2‘,6,6‘-tetrabromobisphenol A (TBBPA) as test compound, targeting the thyroid hormone (TH) signal. The post-implant model showed inactivation of the pluripotent markers and activation of the neuroectodermal markers. The preimplant model resulted high responsive and sensitive to the embryotoxic effect of the tested compounds. The TBBPA was selected to test the potential effects of on viability and neuroectodermal differentiation, assessed through colorimetric and cell-based high-content screening methods establishing sub-toxic (20 μM) and toxic (40 μM) doses. A high-throughput gene expression array-based analysis showed a prompt response of the in vitro testing platform to TBBPA treatment. A rescue experiment exploiting a pan-thyroid receptor (pan-TR) inhibitor (1-850) showed that the effects of TBBPA on RESCs was blocked, demonstrating its activity through TRs.

**Discussion:**

The RESCs-based platform allowed reproducible, robust and highly predictable results, thanks to the coupling of RESCs with high-throughput technologies. These results support the possible use of RESCs-based models as a screening platform for developmental toxicity testing to reduce the number of animals currently used for this aim.

## 1 Introduction

The increasing impact of environmental pollution on human and animal health, with a focus on new persistent organic pollutants and endocrine disruptors, highlights the need for reliable *in vitro* toxicology models to implement early-stage safety assessment, in compliance with the requirements of the regulatory agencies. Actually, to date a consensus is still lacking on the standard *in vitro* tests, or test combinations, which can safely predict the toxic potential of compounds of interest on prenatal development. Three different *in vitro* tests, namely limb mesenchyme micromass culture, rat whole embryo culture (WEC), and mouse embryonic stem cell test (mEST), were proposed to the European Centre for the Validation of Alternative Methods (ECVAM) as alternatives to *in vivo* counterparts. ECVAM assessment deemed both WEC and mEST suitable for predicting the developmental toxicity of chemicals, and the two tests have thus been validated. Specifically, the mEST evaluates the differentiation potential of mouse embryonic stem cells to beating cardiomyocytes while exposed to different concentrations of the compound of interest, allowing the detection of the respective IC50. In addition, the IC50 for mESC and 3T3 cell line (mouse embryonic fibroblasts) viabilities are determined as additional endpoints concurring in the classification of the test compound as a non-, moderate or strong embryotoxicant (Barrier et al., 2011). The main limitations associated with this test are related to the analysis of a specific lineage, the use of cell lines with a low predictive power, and a poor translationality due to the use of mouse-derived cells, while the respective *in vivo* studies are mostly carried out in rats. On the other hand, limitations associated with the exploitation of *in vivo* studies, even though animal models have been regarded as the gold standard for toxicological studies, are well known, being linked mainly with the low throughput, high-cost, labor-intensive, time-consuming and ethical implications characterizing these tests. The most recent (July 2024) report on the use of animals for scientific purposes in the Member States of the European Union and Norway in 2022 (https://circabc.europa.eu/ui/group/8ee3c69a-bccb-4f22-89ca-277e35de7c63/library/051e5787-7746-46cf-8a0d-310f84fd1900/details) indicates that 13% of all animals (13% corresponding to 1.1 million) were used for regulatory studies, of which, 13.8% (corresponding to 151,932 animals) were used for industrial chemical testing, mainly ecotoxicology and developmental toxicology. Prenatal developmental toxicity study testing is regulated by the OECD 414 guideline (https://www.oecd-ilibrary.org/test-no-414-prenatal-development-toxicity-study_5lmqcr2k7ns8.pdf?itemId=/content/publication/9789264070820-en&mimeType=pdf) and comprises the study of the pre-implantation, post-implantation and organogenesis phases, requiring a minimum of 60 pregnant females, usually rats, for each compound to be tested. The huge numbers of animals required for these tests raises inevitable ethical and economic issues, emphasizing the urgency for the development of alternative *in vitro* methods with the aim to replace, or at least reduce, the number of animals needed, in line with the 3Rs principle (Reduction, Replacement, Refinement, https://www.nc3rs.org.uk/who-we-are/3rs). Efforts to validate novel alternative methods characterized by high predictive levels as well as high standardization and translationality (Augustine-Rauch et al., 2016) have been made with non-mammalian species such as zebrafish (Piña et al., 2018) and *Drosophila* (Fukunaga and Kondo, 1985), or by exploiting immature cells for the investigation of specific developmental stages (Toimela et al., 2017). On the other hand, the joint use of advanced culture conditions, microfluidics and omics approaches is rapidly evolving, but these systems still lack the standardization required in a regulatory framework (Rico-Varela et al., 2018). Recently, “stem cell toxicology” emerged as a promising approach to conjugate the need of robust, high-throughput, species-specific approach, in compliance with the regulatory requirements (Fritsche et al., 2021). Our laboratory developed a rat embryonic stem cell line (RESC), which has been fully characterized at the different stages of culture, comprising the 3D stages of embryoid bodies and clusters (Fernández et al., 2011). Most interestingly, we have succeeded in deriving RESC standard 2D adherent cell cultures from the 3D stages, which can be maintained in a pluripotency state, thus mimicking the embryonal pre-implantation stage, or can be guided toward differentiation, thus accounting for the embryonal post-implantation stage (Fernández et al., 2020). The RESC cell line can be used as an easy to use, reproducible and standardized test platform, allowing a flexible analysis approach comprising the most widely used high-content technologies as an alternative *in vitro* platform for the screening of the developmental toxicity of compounds of interest in pre- and post-implantation-mimicking conditions. The present study has the aim to validate the use of a RESC-based platform as a reduction/replacement tool for *in vivo* developmental toxicity testing. We used three different molecules, known for their possible embryotoxic and teratogen effect: the retinoic acid (RA), the perfluorooctanic acid (PFOA), and the 2,2′,6,6′-tetrabromobisphenol A (TBBPA). We then selected the TBBPA as test compound, which is a well-characterized anthropogenic environmental pollutant widely employed as a brominated flame retardant (Covaci et al., 2009), to test its effect on the post-implant model and investigate its mechanism of action. TBBPA is a well-known endocrine disruptor (ED) known to interfere with the thyroid hormone (TH) pathway, supposed to act through the direct binding to the thyroid hormone receptors (TRs) (Zhu et al., 2018).

## 2 Materials and methods

### 2.1 Cell culture

Single rat embryonic stem cells (RESC-SCs) were derived from E4.5 Sprague-Dawley rats at the blastocyst stage as previously described (Fernández et al., 2011). The RESC-SCs were maintained in SCML medium without mitogens (no LIF, no bFGF) and seeded on 0.1% gelatin solution (EmbryoMax, Merck-Millipore Burlington, MA, USA) (Supplementary Material S1 for the full medium composition).

### 2.2 Cell treatments

Retinoic acid (RA) was tested at different concentrations in the pre-implant model (0.25, 0.5, 1, 2, 4, 8, 16, 32, 64, 128, 256, 512 µM), as well as PFOA (10, 20, 40, 80, 160, 200, 280, 320, 640, 1,280 µM).

TBBPA (Sigma-Aldrich) was used at different concentrations (1,200, 640, 320, 160, 80, 40, 20, 10 µM) with or without the RA treatment (1 μM, all-*trans* RA, CAS N. 302-79-4, Sigma-Aldrich) to induce the neuroectoderm lineage differentiation. In the long-term experiments (3, 5, 10 DIVs), selected dosage of TBBPA (20 and 40 µM) and RA were added to the culture medium every 48 h.

In an independent set of experiments, the thyroid hormone receptor antagonist 1-850 (Cayman Chemical, Ann Arbor, MI, USA) was used at a concentration of 10 µM to block the TBBPA effect, as described in previous studies (Fernández et al., 2020).

### 2.3 MTT assay

To assess the viability of the RESC-SCs treated with different concentrations of RA, PFOA, and TBBPA, we used the indirect MTT colorimetric assay, recognized in Good Laboratory Practice guidelines as the gold standard for the evaluation of *in vitro* toxicity, using a standardized protocol (Supplementary Material S1 for the full procedure description).

This assay indirectly semi-quantifies cell viability, using the production of the formazan salts by active mitochondria, and is therefore biased due to the impossibility of distinguishing between dead cells and living cells with impaired mitochondria.

### 2.4 Immunocytochemistry and cell-based high-content screening imaging and image analysis

Immunocytochemistry was performed to analyze the nestin expression, while nuclear staining was used to mark the nuclei and perform the cell death assay using a cell-based high-content screening (HCS) platform (Supplementary Material S1 for the technical details) within 24 h (Gomes et al., 2010). Hoechst-stained nuclei are represented in gray scale when single staining is showed in the pictures to increase the contrast and the image quality, while RGB color images were used when double stainings are presented.

Immunocytochemistry-processed 96-well culture plates were analyzed via cell-based HCS (Cell Insight™ CX5, Thermo Fisher Scientific). The HCS machine and related software allow the acquisition of each entire well included in the experiment, avoiding the operator-dependent bias of choosing the representative field to analyze. Acquisitions were performed using a ×10 objective and the LED lights for the excitation corresponding to the fluorochromes used. The software also allows a standardization of the automatic analysis process based on the fluorescence signals. To quantify cell death, the nuclear morphology was analyzed, using the “compartmental analysis” algorithm. The software recognizes each single nucleus as an object using the Hoechst staining, measuring the number of condensed nuclei in each field and well based on size and fluorescence intensity, and comparing this to the total number of cells per well to give the percentage of cell death per well.

To analyze the differentiation markers, a cytoplasmatic region of interest (ROI) can be created using the same algorithm. This ROI allows selection of a ring-shaped area with its internal perimeter on the border between the nucleus and cytoplasm and its external perimeter within the cytoplasm, with a fixed distance between the two perimeters giving the thickness of the ring. For each cell, the software measures the fluorescence intensity (in arbitrary units, A.U.), and pre-sets a positivity threshold to calculate the percentage of marker-positive cells (on total cell number) per well.

### 2.5 RNA isolation, reverse transcription and semi-quantitative real-time PCR

The gene expression studies were performed using rt-PCR, while total RNA isolation was carried out using the RNeasy Micro Kit (Qiagen, Milan, Italy) and quantified by Nanodrop 2000 spectrophotometer (Thermo Fisher Scientific). cDNA synthesis was performed using the iScriptTMcDNA Synthesis Kit (Bio-Rad, Hercules, CA, USA) following the manufacturer’s instructions, using the same quantity of RNA for all the samples. All the details and the list of primers used for the mRNA quantification are included in Supplementary Material S1.

### 2.6 qPCR arrays

To prepare the real-time qPCR array, total RNA isolation from RESCs was performed using the RNeasy Micro kit (Qiagen) according to the manufacturer’s instructions. RNA was quantified by Nanodrop 2000 spectrophotometer (Thermo Fisher Scientific) and cDNA produced using the RT^2^ First Strand Kit (Qiagen) according to the manufacturer’s instructions.

The Rat Cell Lineage Identification (PARN-508ZD, Qiagen) and Rat Molecular Toxicology PathwayFinder PCR arrays (PARN-401ZD, Qiagen) (Supplementary Materials S2, S3 for the complete list of all analyzed genes) were used to analyze the impact of TBBPA exposure on stem cell viability and differentiation. The RT^2^ SYBR Green qPCR Mastermix (Qiagen) was used for the reaction, following the manufacturer’s instructions for the thermal cycles.

The online software GeneGlobe (Qiagen) was used to analyze the qPCR array data and produce the graphical representations, selecting GAPDH and β-actin as housekeeping genes for the data normalization, as automatically indicated by the software from the different housekeeping genes available. A cut-off value of Cq = 37 was selected for non-expressed genes.

All arrays passed the quality tests which aimed to verify the reproducibility between different plates, test the quality of the PCR reaction, and assess the absence of genomic DNA contamination. Data was analyzed as ∆Cq (∆Cq = Cq_gene_–Cq_HK_) to build the clustergram, and as a “fold of regulation” compared to a control group. The differentially expressed genes were used as inputs for the pathway enrichment analysis through the GeneCodis software (v 4.0) using the KEGG algorithm.

### 2.7 Statistical analysis

For morphological and colorimetric readouts and for gene expression analysis performed by qPCR, all presented data derive from a minimum of three independent experiments, while the number of replicates included in each experiment is indicated in each figure legend. The Prism software (GraphPad, v. 10) was used for statistical analyses and to prepare the graphs. Data in the histograms are reported as mean +SEM. Student’s t-test, one-way ANOVA and Dunnett’s *post-hoc*, two-way ANOVA and Fisher’s LSD *post-hoc*, were used for the analysis, as indicated for each graph in the related figure legend. Results were considered significant when the possibility of their occurrence as a result of chance alone was <5% (p < 0.05).

For gene expression readouts performed by PCR arrays, three independent samples were included in each experimental group. Data were analyzed and represented by GeneGlobe online software (Qiagen), considering p < 0.05 as the cut-off value for statistical significance.

All the data used to generate the graphs and statistical analysis are available from the indicated data repository.

## 3 Results

### 3.1 Modeling the embryonic pre- and post-implantation stage: RA-mediated neuroectodermal differentiation

**FIGURE 1:**
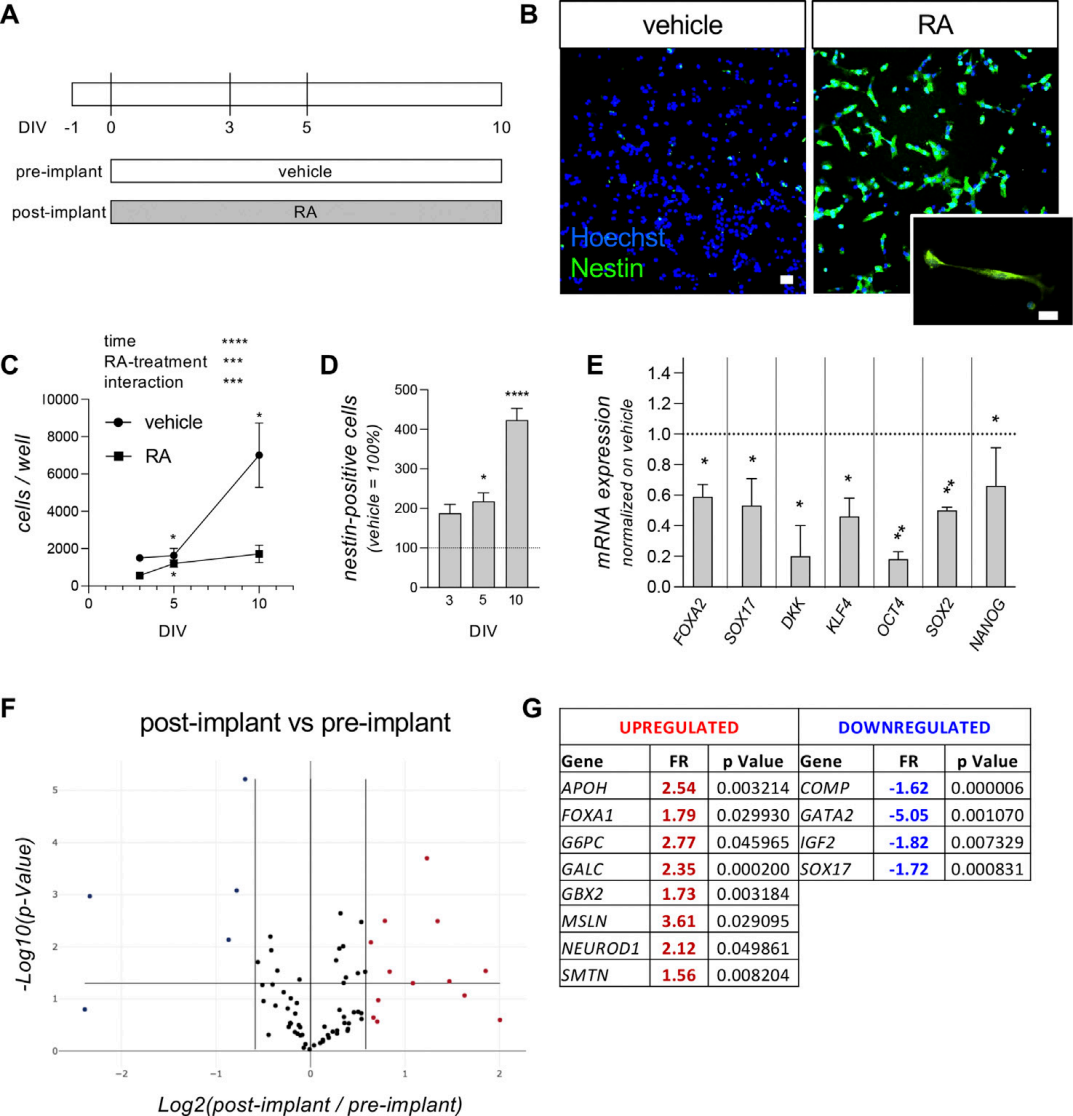
Pre- and post-implant models. **(A)** Experimental protocol. RESC-SC were cultured in standard medium (vehicle) to mimic the pre-implant condition, preserving the pluripotency state, or in presence of retinoic acid (RA; 1 µM) to mimic the post-implant condition, inducing differentiation towards the neuroectodermal lineage. Cultures were treated with vehicle (pre-implant) or RA (post-implant) 24 h after seeding (0 DIV), and neuroectodermal differentiation was analyzed at 3, 5 and 10 DIV, quantifying the percentage of nestin-positive cells in the entire culture using cell-based HCS. Gene expression analysis by qPCR and qPCR array were performed at 3 DIV. **(B)** Representative images of pre-implant (vehicle-treated) and post-implant (RA-treated) cultures at 10 DIV. Cells were stained for nestin (green), with the addition of Hoechst nuclear dye (blue). The panel shows HCS images acquired using a ×10 objective, while the higher-magnification image was acquired using a standard epifluorescence microscope with a ×40 objective. Scale bar: 10 µm. **(C)** Graph shows the total number of cells per well analyzed by HCS, using Hoechst nuclear staining to count every single cell present in each well of each experimental group. Analysis was performed at 3, 5 and 10 DIV in cultures treated with vehicle (pre-implant model) or RA (post-implant model). **(D)** Graph shows the percentage of nestin-positive cells at 3, 5 and 10 DIV. Data are shown as the RA-treated group (post-implant model) compared to the vehicle-treated group (pre-implant model; indicated as a dotted line at y = 100) at each time point. **(E)** Graph shows the gene expression of the indicated genes (*FOXA2*, *SOX17*, *DKK*, *KLF4*, *OCT4*, *SOX2*, *NANOG*) at 3 DIV. Data are represented as the RA-treated group (post-implant model) normalized on the vehicle-treated group (pre-implant model; indicated as a dotted line at y = 1.0) at each time point. **(F, G)** Graph shows the volcano plot **(F)** of the entire gene set analyzed with the “Cell Lineage Identification” PCR array (PARN-508ZD, Qiagen). Each dot represents a single analyzed gene (average value of three replicates) and the position in the graph shows the direction and the magnitude of the regulation. Upregulated genes are indicated by red dots, while downregulated genes are indicated by blue dots, considering the cut-off regulation of 1.5 (vertical lines). Genes are considered significantly regulated if p < 0.05 (horizontal line). The significantly regulated genes, their fold regulation (FR) values and p values are shown in the corresponding tables **(G)**. Statistical analysis. Each point **(C)** or column **(D, E)** represents the mean of the replicates (n = 3–5) + SEM. Two-way ANOVA was used as statistical test for the data shown in graph **(C)**. Results of the analysis are shown in the graph (***p < 0.001; ****p < 0.0001). Asterisks within groups represent differences between two consecutive time points (*p < 0.05). Student’s t-test was used to analyze the data shown in graphs **(D)** and **(E)**. Asterisks represent significant differences between post-implant (RA-treated) and pre-implant (vehicle-treated) groups (*p < 0.05; **p < 0.01; ****p < 0.0001).

We previously demonstrated that the RESC cell line, cultured as a standard bidimensional layer of single cells, expresses pluripotency markers and can be efficiently directed toward neuroectoderm maturation following RA treatment (Fernández et al., 2020). The pre-implant model was then defined as the culture treated with vehicle, preserving the pluripotent state of the stem cell, while the treatment with RA (1 µM) which induced neuroectodermal differentiation was defined as the post-implant model (Figure 1A). Neuroectodermal differentiation is also proven by the expression of the neuroectodermal marker nestin detected by immunocytochemistry (Figure 1B). We further characterized this model using both morphological and molecular techniques. The pre- (vehicle-treated) and post-implant (RA-treated) cultures were initially analyzed for proliferation and differentiation at different time points (3, 5 and 10 days *in vitro*, DIV), quantifying cell proliferation by counting the total number of cells per well at each considered time point as the cell number per well using HCS technology, thus counting all the cells in each well. The data differed significantly for the variables time, RA-treatment and their interaction (two-way ANOVA; time, F (1.110,6.662) = 14.55, p = 0.00065; RA-treatment, F (1,8) = 13.52, p = 0.0063; interaction, F (2,12) = 7.634, p = 0.0073). The vehicle-treated group showed an increase in cell number over the entire time period considered (Fisher’s LSD *post-hoc*, 3 vs. 10 DIV, p = 0.0381; 5 vs. 10 DIV, p = 0.0462), while the RA-treated group showed a slight increase in cell number in the first period only (3 vs. 5 DIV, p = 0.0176), proving that the pre-implant model is characterized by proliferating cells, while the post-implant model is composed of a high prevalence of viable post-mitotic cells (Figure 1C). In line with this result, the percentage of nestin-positive cells in post-implant compared to pre-implant cultures gradually increases after 5 DIV (Student’s t-test, p = 0.0366) and 10 DIV (p < 0.0001) (Figure 1D). Gene expression analysis by qPCR indicated that the RA treatment (post-implant model) significantly downregulates the expression level of pluripotency genes (Student’s t-test; *OCT4*, p = 0.0181; *NANOG*, p = 0.0119; *SOX2*, p = 0.0016; *KLF4*, p = 0.0326, Figure 1E) compared to vehicle-treated (pre-implant model) cultures, as well as significantly decreasing the expression of endoderm (*SOX17*, p = 0.0167; *FOXA2*, p = 0.0261) and mesoderm (*DKK*, p = 0.0110) markers. The pre- and post-implant models were also analyzed using the PCR array technology, quantifying the gene expression of 84 genes involved in the differentiation of pluripotent stem cells in the three different germ layers (Figures 1F,G). Three samples per experimental group were analyzed, comparing the RA-treated (post-implant model) with the vehicle-treated (pre-implant model) cells, after 3 days of RA exposure (Supplementary Material S2 for the full list of analyzed genes). A 1.5-fold difference (FR, fold regulation) was established as significant, but only if the statistical significance level of p < 0.05 was reached. RA exposure produced an upregulation of eight genes (*APOH*, *FOXA1*, *G6PC*, *GALC*, *GBX2*, *MSLN*, *NEUROD1*, *SMTN*) and a downregulation of four genes (*COMP*, *GATA2*, *IGF2*, *SOX17*).

### 3.2 The pre-implant model is highly responsive and sensitive to the embryotoxic effect induced by RA, TBBPA and PFOA

To mimic the OECD flowchart for developmental toxicology studies, we first tested the three compounds, RA, TBBPA and PFOA in the pre-implant model. RESCs were exposed to different concentrations of the three compounds, and acute toxicity (24 h exposure) was analyzed using the MTT biochemical assay and the HCS-based cell count.

**FIGURE 2:**
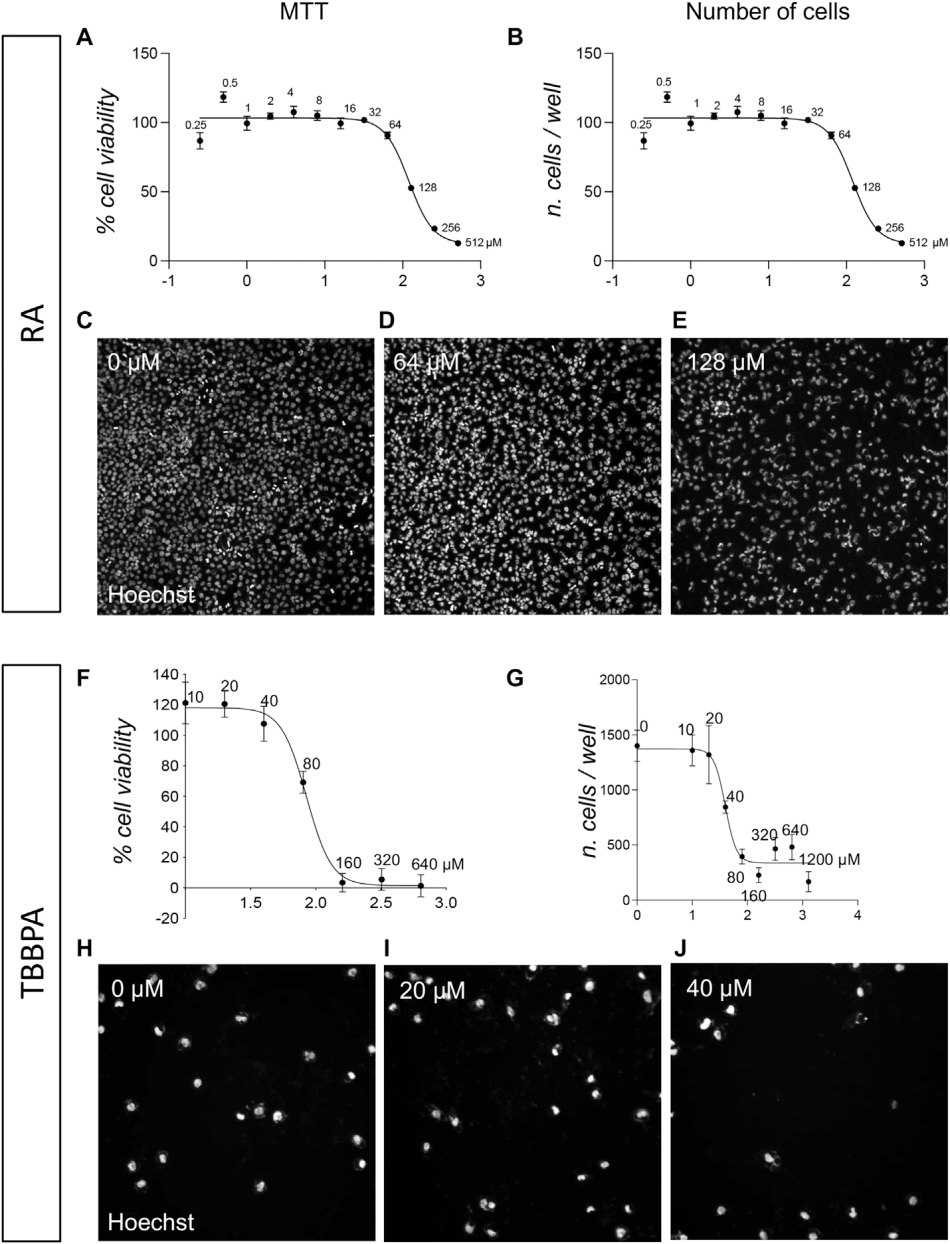
Acute toxicity (24 h) on pre-implant cultures of RA and TBBPA. **(A)** Graph shows the MTT cell viability assay of pre-implant cultures treated with different doses of RA (0.25, 0.5, 1, 2, 4, 8, 16, 32, 64, 128, 256, 512 µM), represented as a percentage of the control group (0 µM = 100%). **(B)** Graph show the cell-based HCS analysis of pre-implant cultures treated with different doses of RA (0.25, 0.5, 1, 2, 4, 8, 16, 32, 64, 128, 256, 512 µM) expressed as number of viable cells per well. **(C–E)** Representative HCS images of pre-implant cultures treated with 0 µM **(C)**, 64 µM **(D)**, 128 µM **(E)** RA and stained using Hoechst nuclear dye. **(F)** Graph shows the MTT cell viability assay of pre-implant cultures treated with different doses of TBBPA (10, 20, 40, 80, 160, 320, 640 µM), represented as a percentage of the control group (0 µM = 100%). **(B)** Graph show the cell-based HCS analysis of pre-implant cultures treated with different doses of TBBPA (0, 10, 20, 40, 80, 160, 320, 640, 1,200 µM) expressed as number of viable cells per well. **(H–J)** Representative HCS images of pre-implant cultures treated with 0 µM **(H)**, 20 µM **(I)**, 80 µM **(J)** TBBPA and stained using Hoechst nuclear dye. Each point **(A, B, F, G)** represents the mean of n = 5 replicates +SEM.

The MTT viability assay was performed according to the manufacturer’s instructions to calculate the IC_50_, which resulted to be for the RA 119.9 µM (best fit; 95% CI 101.2–149.3 µM; Figure 2A). While the MTT assay is the standard test in Good Laboratory Practice validated assays for assessing acute toxicity *in vitro* (Coecke et al., 2005), it consists of an indirect measurement of cell viability through mitochondrial metabolism, prompting us to perform a direct evaluation of cell viability in parallel via the HCS analysis of nuclear morphology. This technique consists of an imaging-based quantification of the percentage of apoptotic cells, considering the entire culture at single-cell resolution. As described in the methods section, the HCS software detects the Hoechst staining, recognizing each individual nucleus as an object. Using a Boolean intersection of two thresholds (area/size and fluorescence intensity), the percentage of condensed nuclei (low area/size, high dye intensity) can be analyzed in all experimental groups (about 1,500 cells/well in the control group). Apoptosis leads to the formation of condensed nuclei as the last step of the process, followed by the detaching of the apoptotic cell or its remaining fragments. Analyzing the percentage of viable cells and counting the total cell number per well therefore gives a complete overview. The HCS quantification of viable cells resulted in the identification of the same IC_50_ value for the RA, 119.9 (best fit; 95% CI 101.2–149.3 µM; Figure 2B). Representative pictures are included in Figures 2C–E.

For the TBBPA, the MTT viability assay was performed according to the manufacturer’s instructions to calculate the IC_50_, which resulted to be 84.84 µM (best fit; 95% CI 77.63–92.72 µM) (Figure 2F). The highest concentration (1,200 µM) was excluded from the IC50 calculation due to its very low absorbance value, which resulted in an unreliable relative absorbance value once the blank value had been subtracted. A HCS-based count of the remaining cells in the well revealed that at a concentration of 40 μM, about 50% of cells were already detached due to cell death induction by TBBPA exposure (IC_50_ calculated on the cell number values, 39.74 µM; best fit, 95% Cl 32.03–47.93 µM; Figure 2G). Moreover, the HCS analysis based on the quantification of condensed nuclei, showed that the quantification of the viable cell percentage, calculated by subtracting the percentage of dead cells from the total number of cells, is more sensitive than MTT assay, with an IC50 of 58.84 µM using this morphology-based method (best fit; 95% CI 51.92–66.34 µM) (Supplementary Figure S1). Representative pictures are included in Figures 2H–J.

**FIGURE 3:**
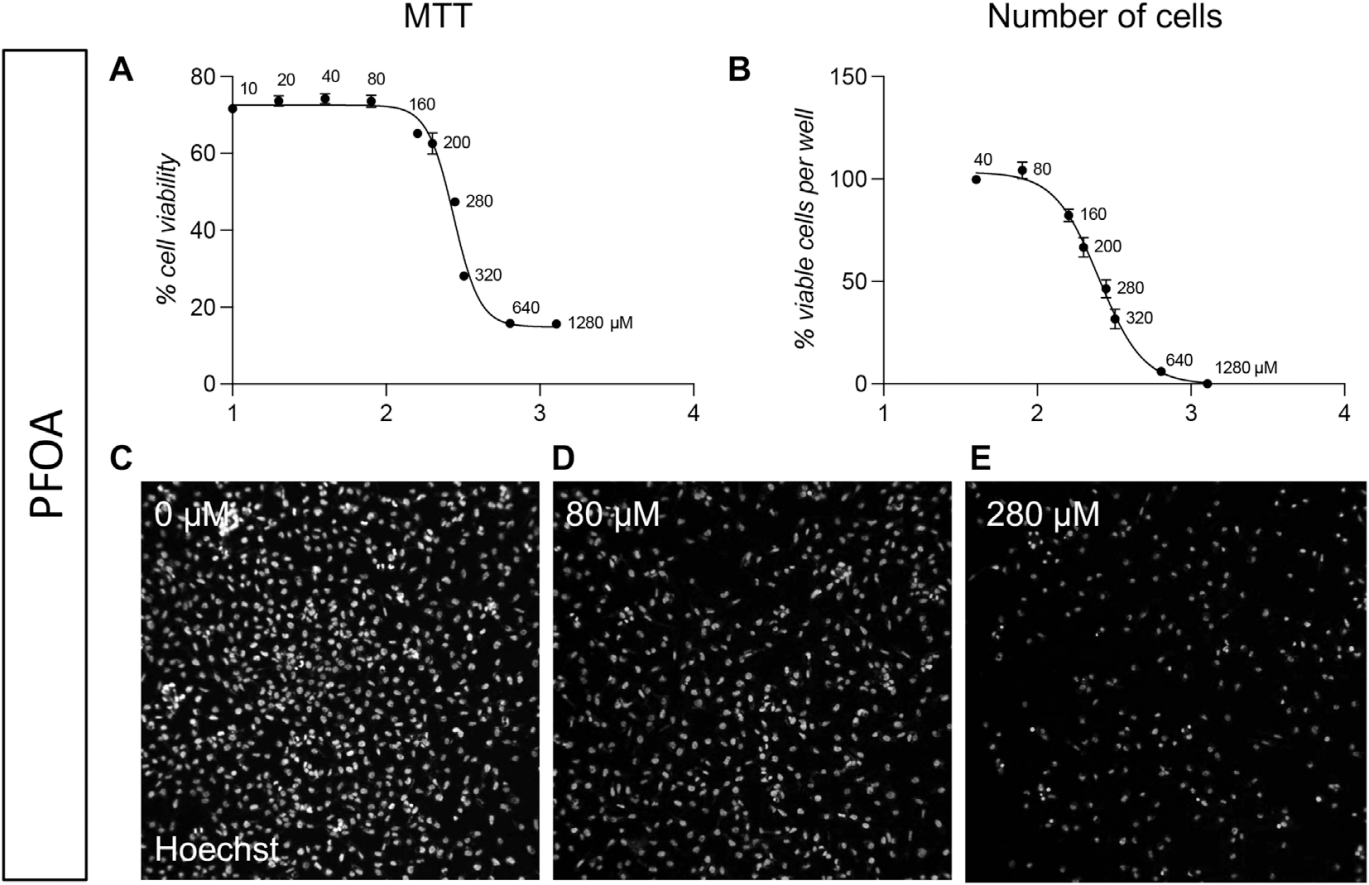
Acute toxicity (24 h) on pre-implant cultures of PFOA. **(A)** Graph shows the MTT cell viability assay of pre-implant cultures treated with different doses of PFOA (10, 20, 40, 80, 160, 200, 280, 320, 640, 1,280 µM), represented as a percentage of the control group (0 µM = 100%). **(B)** Graph show the cell-based HCS analysis of pre-implant cultures treated with different doses of PFOA (40, 80, 160, 200, 280, 320, 640, 1,280 µM) expressed as number of viable cells per well. **(C–E)** Representative HCS images of pre-implant cultures treated with 0 µM **(C)**, 80 µM **(D)**, 280 µM **(E)** PFOA and stained using Hoechst nuclear dye. Each point (AB, Each point **(A, B)** represents the mean of n = 5 replicates +SEM.

We also tested a third molecule, the PFOA, based on its putative embyotoxic effect. The MTT analysis showed an IC50 of 274.2 µM (best fit; 95% CI 261.2–286.6 µM; Figure 3A), and a similar result was obtained by the HCS-based analysis, with an IC50 calculated as 251.6 µM (best fit; 95% CI 231.8–273.8 µM; Figure 3B). Representative pictures are included in Figures 3C–E.

### 3.3 TBBPA as a reference compound for the set-up of an *in vitro* screening platform for endocrine disruptor chemicals

The TBBPA was selected as a test-molecule for the test on the post-implant model, for its putative mechanism mediated by the TRs.

**FIGURE 4:**
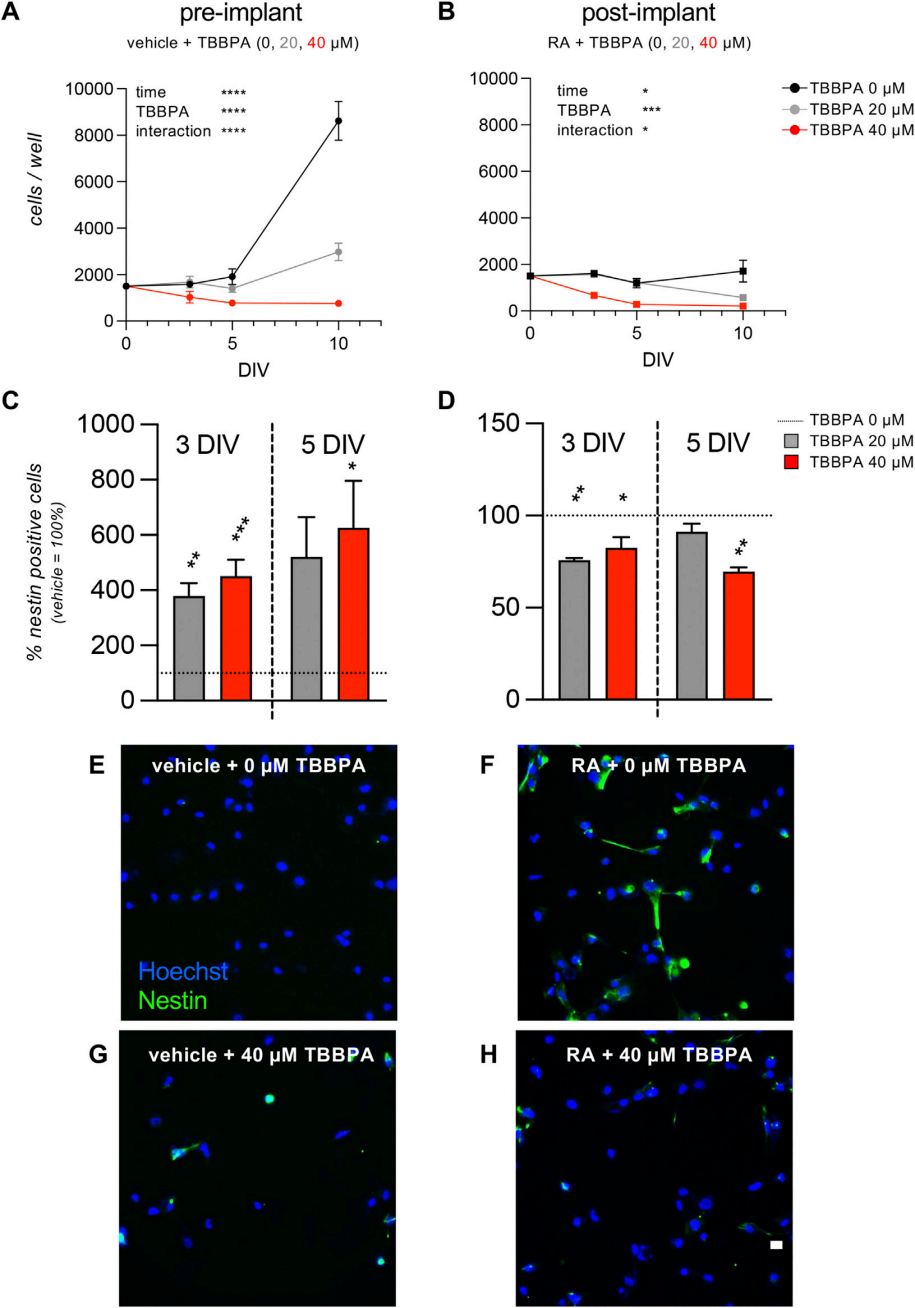
Time course on pre- and post- implant cultures. **(A, B)** Graphs show the HCS analysis of pre-implant (vehicle, **A**) and post-implant (RA, **B**) cultures treated with three different doses of TBBPA (0, 20, 40 µM), expressed as cell number per well, measured at 0, 3, 5, and 10 DIV of exposure. **(C, D)** Graphs show the HCS analysis of pre-implant (vehicle, **C**) and post-implant (RA, **D**) cultures treated with three different doses of TBBPA (0, 20, 40 µM), expressed as percentage of nestin-positive cells, measured at 3 and 5 DIV of exposure. Results are normalized on the control group (0 µM = 100%, horizontal dotted line) of the same experiment. **(E–H)** Representative images of pre-implant (vehicle-treated, **E**, **G**) and post-implant (RA-treated, **F**, **H**) cultures treated with 0 µM TBBPA (control group, E, F) or 40 µM TBBPA **(G,H)**. Scale bar: 10 µm. Statistical analysis. Each point **(A, B)** or column **(C, D)** represents the mean of the different replicates (n = 3–5, **A**, **B**; n = 4, **C**, **D**) + SEM. Two-way ANOVA was used to analyze the data shown in graphs **(A)** and **(B)**. Results of the analysis are shown in the graphs (*p < 0.05; ***p < 0.001; ****p < 0.0001). One-way ANOVA followed by Dunnet’s *post-hoc* was used to analyze the data shown in graphs **(C)** and **(D)**. Asterisks represent significant differences between post-implant (RA-treated) and pre-implant (vehicle-treated) groups (*p < 0.05; **p < 0.01; ****p < 0.0001).

The acute toxicity analysis was used to select the sub-toxic and toxic doses to be tested in the differentiation/toxicity analysis in the pre- and post-implant models. Twenty µM was selected as the sub-toxic concentration, since the 20 µM TBBPA-exposed group showed a viability of 100% in the MTT assay, with no statistical differences for the quantification of % condensed nuclei or total count of cell/well number. On the other hand, 40 µM resulted as being the first toxic concentration, with a cell viability decrease of around 30%–50%, resulting in a mild acute toxicity. Representative images of pre-implant cultures exposed to 0, 20, 40, and 80 µM TBBPA for 24 h and stained with Hoechst are shown in Figures 2D–G. We used the selected doses to test the effect of the selected sub-toxic and toxic doses of TBBPA on the RESC-based pre- and post-implant models, exposing the vehicle-treated (pre-implant model) and RA-treated (post-implant model) cultures to 0, 20 and 40 µM of TBBPA. Cells were fixed at 3, 5 and 10 DIV, stained for nestin expression and Hoechst nuclear staining, and analyzed by HCS. The pre-implant model culture showed an effect on cell number mediated by time, TBBPA treatment, and the interaction between the two variables (two-way ANOVA; time, F (1.585,8.451) = 50.71, p < 0.0001; TBBPA, F (2,10) = 33.97, p < 0.0001; interaction, F (6,16) = 30.12, p < 0.0001). As expected, the control group, not exposed to TBBPA, showed an increase in cell number due to time in culture (multiple comparison of different time points within the same treatment; 3 vs. 10 DIV, p = 0.0075; 5 vs. 10 DIV, p = 0.0250). The TBBPA group treated with the sub-toxic dose showed a smaller increase in cell number (5 vs. 10 DIV, p = 0.0275), while the toxic dose blocked any increase in cell number, resulting in no significant differences (Figure 4A). The post-implant model also showed an effect on cell number mediated by time, TBBPA treatment and the interaction between the two variables (two-way ANOVA; time, F (1.136,10.23) = 5.141, p < 0.0428; TBBPA, F (2,27) = 12.17, p = 0.0002; interaction, F (6,27) = 2.800, p = 0.0300). While there were no differences in cell number due to time in culture in any of the groups, there was a clear reduction due to the toxic dose of TBBPA (40 µM) at all considered time points (TBBPA 40 vs. 0 μM; 3 DIV, p = 0.0006; 5 DIV, p = 0.0145; 10 DIV, p = 0.0473) (Figure 4B). To analyze the effect on neuroectodermal differentiation, we then quantified the percentage of cells expressing nestin. In the pre-implant model, in which the pluripotency of stem cells is maintained, the TBBPA exposure induced an increase of nestin-positive cells, both after 3 and 5 DIV (3 DIV: one-way ANOVA, F (2,9) = 16.45, p = 0.0010; Dunnett’s post-hoc, 20 μM, p = 0.0036; 40 μM, p = 0.0008; 5 DIV: one-way ANOVA, F (2,9) = 4.654, p = 0.0409; Dunnett’s post-hoc, 40 μM, p = 0.0325) (Figure 4C). On the contrary, in the post-implant model, in which the RA was present in the culture medium triggering the neuroectodermal differentiation, the presence of TBBPA, both as sub-toxic and toxic dose, led to a reduction of nestin-positive cells compared to the 0 µM TBBPA control group (3 DIV: one-way ANOVA, F (2,8) = 8.799, p = 0.0095; Dunnett’s post-hoc, 20 μM, p = 0.0058; 40 μM, p = 0.0148; 5 DIV: one-way ANOVA, F (2,6) = 12.49, p = 0.0073; Dunnett’s post-hoc, 40 μM, p = 0.0044) (Figure 4D). Representative images of pre- and post-implant models exposed to 0 and 40 µM TBBPA at 5 DIV are included in Figures 4E–H. To further analyze the effect of TBBPA on pre- and post-implant models, we then selected the time point of 3 DIV-exposure. This guarantees a robust effect of the molecule, keeping enough cells alive to allow the analysis using morphological and molecular readouts.

### 3.4 Pre- and post-implant RESC-based models to test the differentiation interference and embryotoxicity potential of TBBPA using transcriptomic readouts

**FIGURE 5:**
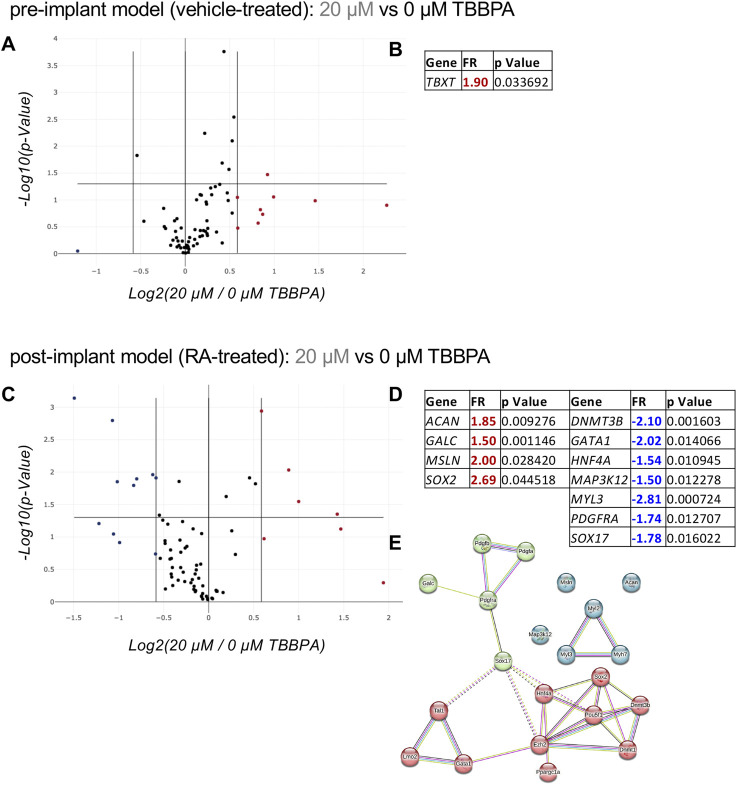
Effect of a sub-toxic dose of TBBPA on RESC-SC differentiation analyzed by the “Cell Lineage Identification” PCR array. **(A, B)** Relative gene expression on pre-implant (vehicle-treated) cultures exposed for 3 DIV to a sub-toxic dose of TBBPA (20 µM) compared to the control group (0 µM TBBPA), represented as a volcano plot **(A)** (PARN-508ZD, Qiagen); the significantly regulated (FR > +1.5; p < 0.05) gene, its fold regulation (FR) value and p value, is included in the table **(B)**. **(C–E)** Relative gene expression on post-implant (RA-treated) cultures exposed for 3 DIV to a sub-toxic dose of TBBPA (20 µM) compared to the control group (0 µM TBBPA), represented as a volcano plot **(C)** (PARN-508ZD, Qiagen); the significantly upregulated (FR > +1.5; p < 0.05, red values) and downregulated (FR < −1.5; p < 0.05, blue values) genes, their fold regulation (FR) values and p values, are included in the table **(D)**, and where used to generate the protein-protein interaction network using the online software STRING **(E)**. Statistical analysis. The PCR array analysis was performed using n = 3 replicates per conditions. In the volcano plots **(A, C)** each dot represents a single gene analyzed, and the position in the graph shows the direction and magnitude of its regulation. Upregulated genes are represented by red dots, while downregulated genes by blue dots, considering the cut-off regulation of 1.5 (vertical lines). Genes are considered significantly regulated if p < 0.05 (horizontal line). The STRING analysis **(E)** was generated using the proteins encoded by the differentially regulated genes in post-implant cultures as input (query proteins), with the addition of 10 proteins from the first shell of interactors (directly interacting with the query proteins). Each circle corresponds to a single protein (node), and different colors represent different clusters (k-means = 3). Lines represent the connection between nodes (edges), generated by four different interaction sources (Textmining, Experiments, Databases, Co-expression) indicated by different colors. Edges between clusters are shown as dotted lines **(E)**.

After the characterization phase demonstrating the effect of the environmental pollutant TBBPA acting as endocrine disruptor on the TH/RA regulated pathway, we developed a molecular biology-based readout to assess its interference with differentiation and embryotoxic potential. The differentiation assay was performed on the pre- and post-implant models using the sub-toxic dose and the exposure time (3 DIV) selected in the previous experiments, using the PCR array technology to quantify the expression of genes involved in the differentiation of pluripotent cells in the mesoderm, ectoderm, and endoderm lineages (RT2 Profiler™ Rat Cell Lineage Identification PCR array, PARN508Z). The array included genes specific for the three germ layers (ectoderm, mesoderm, endoderm), both as progenitors and terminal differentiated cells (Supplementary Material S2). Three replicates were included in each experimental group, and regulations were considered significant when FR ≥ 1.5 with a p < 0.05. In the pre-implant model, the only regulated gene was TBXT (Figures 5A,B), a gene which encodes for the Brachyury protein. In the presence of the differentiation trigger RA as a post-implant model, the sub-toxic TBBPA dose generated a greater perturbation in the gene expression related to differentiation (Figures 5C,D). Four genes were upregulated (ACAN, GALC, MSLN, SOX2) and seven downregulated (DNMT3B, GATA1, HNF4A, MAP3K12, MyL3, PDGFRA, SOX17). An examination of all regulated genes in the protein-protein interaction net generated by the STRING online software, and the addition of ten genes directly linked to the input nodes showed SOX17 to be the key player connecting the three clusters (Figure 5E). Using the same process, we then analyzed the toxic dose (40 µM TBBPA) for its ability to regulate genes involved in the cell death pathways, using the specific qPCR array (RT2 Profiler™ PCR array Rat Molecular Toxicology Pathway Finder, PARN401Z) which includes genes involved in apoptosis, necrosis, DNA damage/repair, oxidative stress, heat shock response, endoplasmic reticulum stress, and other cell death mechanisms (Supplementary Material S3).

**FIGURE 6:**
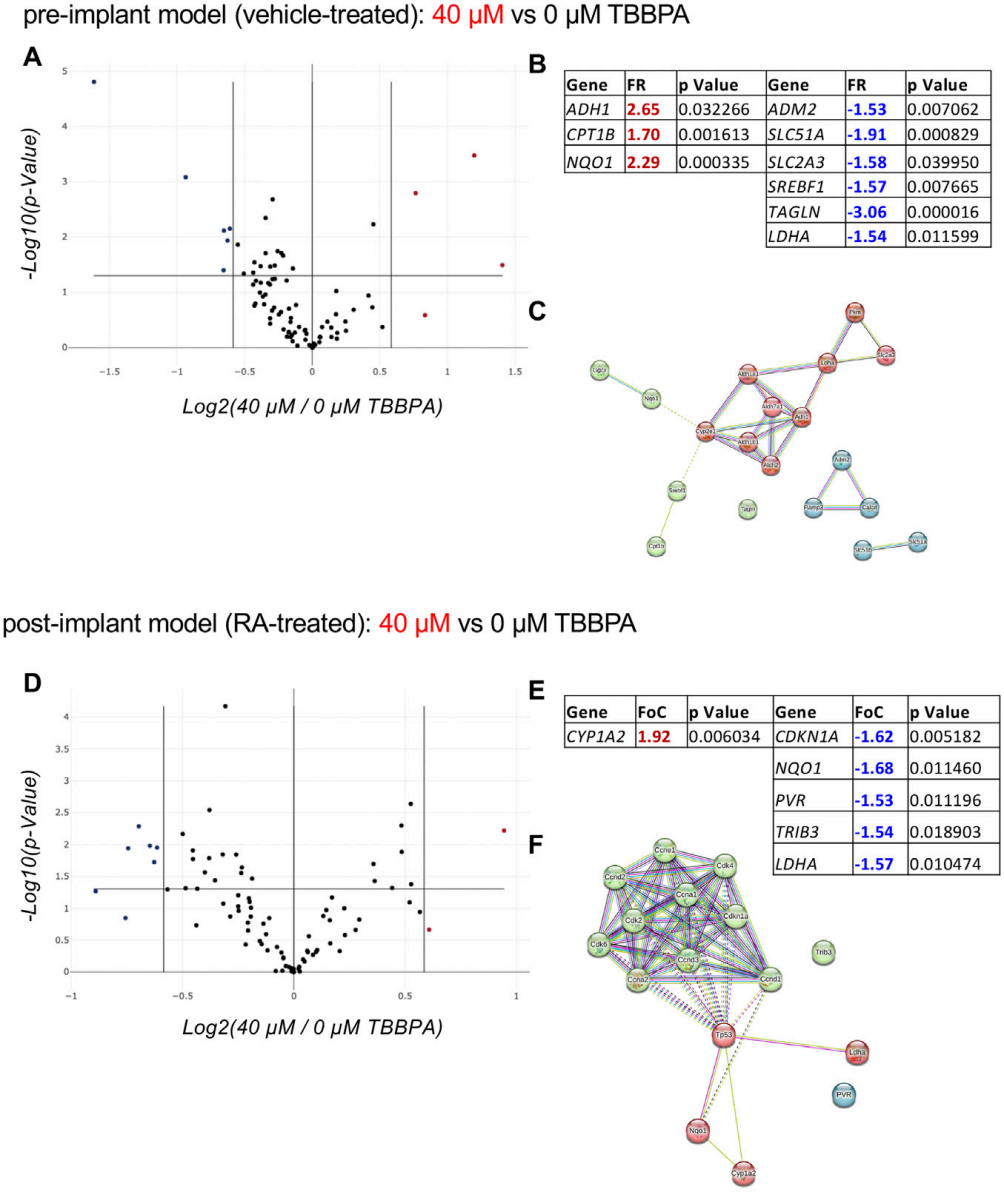
Effect of a toxic dose of TBBPA on RESCs viability. **(A–C)** Relative gene expression on pre-implant (vehicle-treated) cultures exposed for 3 DIV to a toxic dose of TBBPA (40 µM) compared to the control group (0 µM TBBPA), represented as a volcano plot **(A)** (PARN-401ZD, Qiagen); the significantly upregulated (FR > +1.5; p < 0.05, red values) and downregulated (FR < −1.5; p < 0.05, blue values) genes, their fold regulation (FR) values and p values, are included in the table **(B)**, and where used to generate the protein-protein interaction network using the online software STRING **(C)**. **(D–F)** Relative gene expression on post-implant (RA-treated) cultures exposed for 3 DIV to a toxic dose of TBBPA (40 µM) compared to the control group (0 µM TBBPA), represented as a volcano plot **(D)** (PARN-401ZD, Qiagen); the significantly upregulated (FR > +1.5; p < 0.05, red values) and downregulated (FR < −1.5; p < 0.05, blue values) genes, their fold regulation (FR) values and p values, are included in the table **(E)**, and where used to generate the protein-protein interaction network using the online software STRING **(F)**. Statistical analysis. The PCR array analysis was performed using n = 3 replicates per conditions. In the volcano plots **(A, D)** each dot represents a single gene analyzed, and the position in the graph shows the direction and magnitude of the regulation. Upregulated genes are represented by red dots, while downregulated genes are represented by blue dots, considering the cut-off regulation of 1.5 (vertical lines). Genes are considered significantly regulated if p < 0.05 (horizontal line). The STRING analysis **(C, F)** was generated using the proteins encoded by the differentially regulated genes in post-implant cultures as input (query proteins), with the addition of 10 proteins from the first shell of interactors (directly interacting with the query proteins). Each circle corresponds to a single protein (node), and different colors represent different clusters (k-means = 3). Lines represent the connection between nodes (edges), generated by four different interaction sources (Textmining, Experiments, Databases, Co-expression) indicated by different colors. Edges between clusters are shown as dotted lines **(E)**.

In the pre-implant model, three genes were upregulated (ADH1, CPT1B, NQO1) and six were downregulated (ADM2, SLC51A, SLC2A3, SREBF1, TAGLN, LDHA) (Figures 6A,B). Interestingly, by analyzing the STRING protein-protein interaction net with 10 proteins of the first shell of interaction, a clear cluster of the RA metabolism and action emerges (ALDH1A1, ALDH7A1, ALDH1, ALDH1B1, ALDH2, CYPE1, LDHA, PKM, SLC2A3) (Figure 6C), confirming that TBBPA can activate the neuroectodermal differentiation pathway when RA is not present in the culture medium. On the other hand, when RA is present (post-implant model), the toxic dose of TBBPA generates one upregulated gene (CYP1A2) and five downregulated genes (CDKN1A, NQO1, PVR, TRIB3, LDHA) (Figures 6D,E). Only PVR and LDHA are shared by the two models, while the others are regulated in the post-implant model only. The STRING protein-protein interaction net with 10 proteins of the first shell of interaction shows the TP53 gene as the central node (Figure 6F). The heatmap representation of the PCRarray data are included in Supplementary Material S4.

### 3.5 Validation of the RESC-based model through the TBBPA action rescue experiment

**FIGURE 7:**
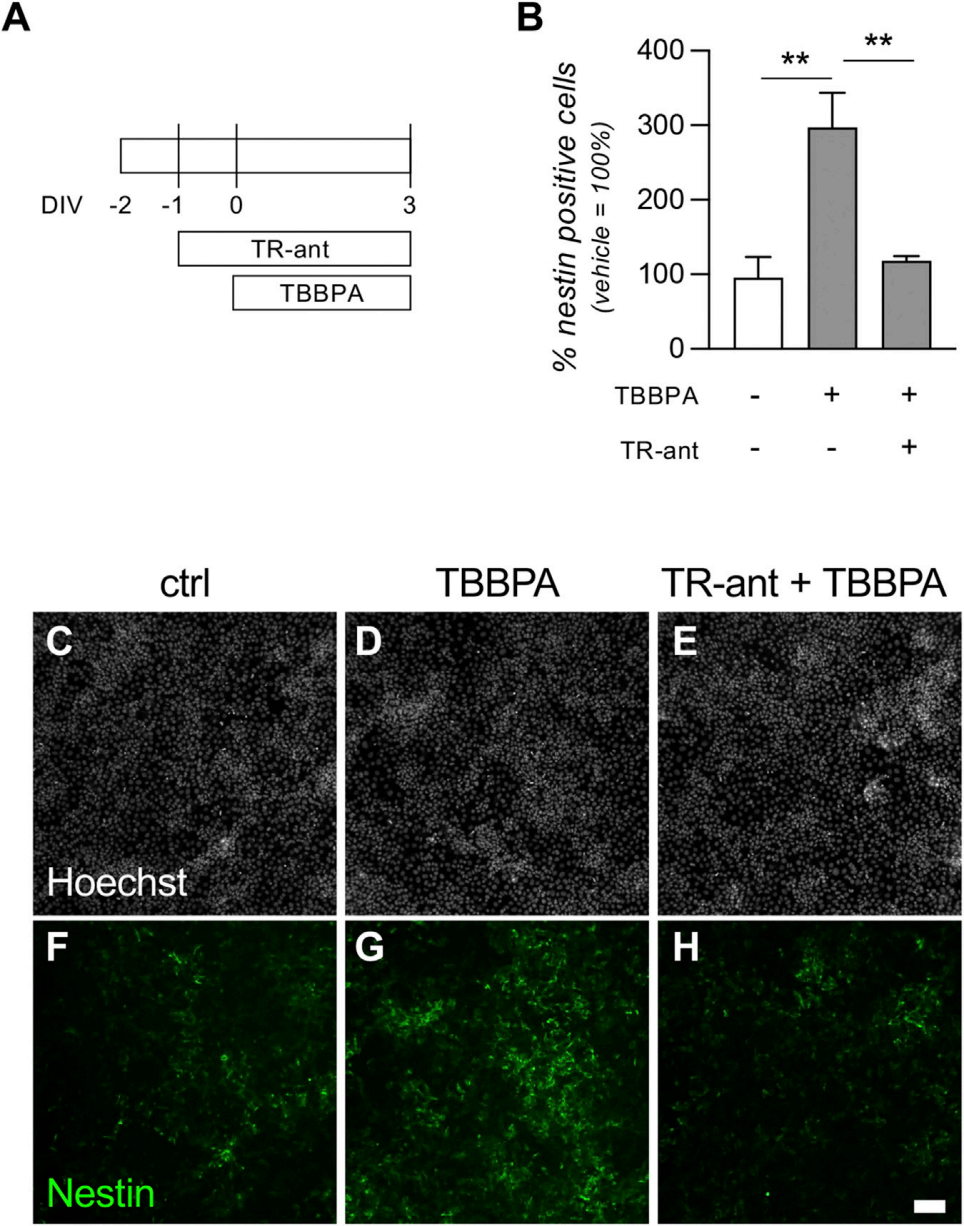
Effect of a thyroid hormone receptor antagonist on TBBPA mechanism of action. **(A)** Experimental protocol. RESC-SC pre-implant cultures were treated with the thyroid hormone receptor antagonist 1-850 (TR-ant) 24 h after seeding (−1 DIV). After a further 24 h (0 DIV), cultures were treated with a sub-toxic dose of TBBPA (20 µM). Neuroectodermal differentiation was analyzed after 3 DIV, quantifying the percentage of nestin-positive cells in the entire culture using cell-based HCS. **(B)** Graph shows the percentage of nestin-positive cells at 3 DIV. Data are represented as a percentage of the control group, which was not exposed to TR-ant nor to TBBPA (white column = 100%). **(C-H)** Representative images of RESC-SC pre-implant control cultures **(C,F)**, treated with TBBPA **(D,G)**, or pre-treated with TR-ant and then exposed to TBBPA **(E,H)**. Scale bar: 50 μm. Statistical analysis. Each column **(B)** represents the mean of the replicates (n = 4) + SEM; analysis was performed by one-way ANOVA followed by Dunnet’s *post-hoc*. Asterisks represent significant differences between post-implant (RA-treated) and pre-implant (vehicle-treated) groups (*p < 0.05; **p < 0.01; ****p < 0.0001).

To validate the *in vitro* platform as cell systems capable of testing endocrine disruptors acting on nuclear receptors, we performed a rescue experiment to demonstrate the direct effect of TBBPA on TRs. To date, the dynamic of TBBPA molecular action is not clear, the current hypothesis being that it directly binds the TRs, acting both as agonist and antagonist, but little evidence is available in the literature (Ghassabian and Trasande, 2018; Miao et al., 2023). In our experiments, we demonstrated its action as a weak agonist, activating the differentiation machinery in pluripotent cells, but blocking neuroectodermal differentiation when triggered by the physiological RA-dependent pathway. To definitively prove that TBBPA action is mediated by TRs, we pre-treated the pre-implant model cultures with a molecular tool acting as strong TR antagonist (1-850, TR-ant). We already described how this compound blocks the RA action on neuroectodermal induction in RESC cultures in a similar way to TBBPA, leading to the hypothesis that they bind the same receptors. After 24 h, we exposed the culture to 20 µM TBBPA, incubating the cultures for 3 DIV prior to processing (Figure 7A). Quantification of the percentage of nestin-positive cells showed a statistically significant difference between groups (one-way ANOVA, F (2,9) = 12.51, p = 0.0025). In cultures treated with TBBPA only, we replicated the previous results, proving that it triggers neuroectodermal differentiation, increasing the percentage of nestin-positive cells compared with the control group (Tukey’s *post-hoc*, p = 0.0035). When cultures were pre-treated with the TR-ant, however, this effect was completely inhibited (TBBPA only vs. TR-ant + TBBPA, p = 0.0073) (Figure 7B). HCS-acquired pictures of cultures treated with vehicle (ctrl), TBBPA only, or both TR-ant + TBBPA, and stained with Hoechst nuclear staining and nestin, are shown in Figures 7C–H.

## 4 Discussion

The increasing need to monitor environmental pollution requires fast and reliable screening methods compliant with OECD guidelines (Fouyet et al., 2024). Endocrine-disrupting chemicals (EDCs) are a severe hazard for human and animal health (Lazofsky and Buckley, 2022), and include many emerging contaminants with high persistence in the environment and a high likelihood of bioaccumulation in the food chain, leading to the dysregulation of hormone secretion, with negative effects for development, growth, reproduction, metabolism, immunity and behaviour. Here, we present a cell system suited to first-line screening for EDC toxicity, replacing at least part of the animal tests required by the OECD 414 guideline for the Prenatal Developmental Toxicity Study. We have described how the cell system based on adherent RESC cultures: i) can be chemically controlled to mimic the molecular and differentiation profile of both pre- and post-implantation embryos, ii) responds in a dose-dependent manner to toxic challenges distinguishing pre- and post-implant stages, and iii) whose toxicity readout can be analyzed using high-content methods. By combining automated microscopy, quantitative image analysis and high computing power, HCS is emerging as a huge step forward for cell culture experiment analysis, dramatically increasing reproducibility and reliability in the profiling of compounds in toxicology studies (Li and Xia, 2019). Our test compound was TBBPA, a contaminant detected in numerous abiotic and biological matrixes. The global TBBPA market reached $1,216.26 million in 2020, and is expected to reach $1,904.60 million by 2028, with a 5% annual growth rate from 2021 to 2028 (https://www.coherentmarketinsights.com/market-insight/tetrabromobisphenol-a-market-5013) (Gregory, 2006).

### 4.1 2D RESCs as pre- and post-implant cellular models

We set *in vitro* cultures to mimic embryonic stem cells at pre- and post-implantation stages, using a protocol which avoids 3D topography, thus allowing routine use of cell-based HCS. An adherent monoculture of embryonic stem cells, able to differentiate into neuroectodermal lineage without multicellular aggregation or co-culture, strongly decreases experimental variability while maintaining the key molecular machinery for lineage induction (Ying et al., 2003). By using rat embryonic stem cells, we were able to mimic both the pre-implant, early blastocyte state, and the post-implant mid-blastula transition and early gastrulation, i.e., the stage for induction of the three germinal layers (ecto- and neuroectoderm, mesoderm, endoderm), thus advancing the cell system based on pluripotent J1 mouse embryonic stem cells (Barrier et al., 2011). The two differentiation stages are well described by the molecular signature of cells at pre- and post-implantation stage. Blastomere compaction in the morula and the formation of the blastocyst characterize the morphogenetic events in pre-implantation development. The pre- to post-implant transition is characterized by epiblast remodeling and gastrulation, in which a main event is endothelial-to-mesenchymal transition (EndMT), leading to formation of the three germinal layers (Kojima et al., 2014). At this stage, RA provides instructive signals through nuclear receptors that bind DNA and directly regulate the transcription of key genes for very early patterning and development of the posterior neuroectoderm, foregut endoderm and trunk mesoderm differentiation (Duester, 2008). Our cellular system is RA-sensitive, as indicated by the drastic downregulation of three genes: GATA2, a pluripotency inducer (Shu et al., 2015), whose transcription is inhibited by RA (Tsuzuki et al., 2004); CAMP, which is also associated with pluripotency (Fritz et al., 2015), and IGF2, which supports the self-renewal of the embryonic stem cell and blastomeres *in vitro* (Yuan and Hong, 2017). RA exposure also leads to a recapitulation of transcriptomic features of this pre-to-post-implant transition, such as the upregulation of EndMT-related genes such as mesothelin (MSLN), an EndMT-promoter in different cell types (Hu et al., 2024). These genes include G6PC- (glucose-6-phosphatase catalytic subunit), which also promotes EndMT in cancer cells (K. Zhu et al., 2022), primitive streak genes such as FOXA1, a pioneer transcription factor during differentiation of embryonic stem cells (Taube et al., 2010), and the upregulation of early germinal layers inducers such as neuronal differentiation 1 (NEUROD1), neuroectodermal inducer (Choi et al., 2020); Gastrulation Brain Homeobox 2 (GBX2), involved in establishing the hindbrain (Waters and Lewandoski, 2006), and smoothelin (SMTN), a protein of the smooth muscle cells (Deruiter et al., 2001).

### 4.2 2D RESCs as a test system for the embryotoxicity effect of RA, PFOA, and the endocrine disruptor compound TBBPA

To test the RESC-based cellular system, we used three different molecules for the acute toxicity on the pre-implant model. The platform resulted highly sensitive and responsive, validating also the HCS-based technique compared to the gold standard MTT assay. The RA, in fact, is the main factor inducing the neuroectoderm differentiation, used to induce the post-implant model. However high dosage of the molecule acts as a well-known teratogen. Our system resulted sensitive as the same level of the more complex and less reproducible model based on mouse embryos (Huang et al., 2005).

TBBPA toxicity starts form 40 µM with a differentiation interference already detected at 20 μM, while the gold standard mESC-based test highlights a 24 h toxicity starting from 150 µM (Yin et al., 2018). We included also a pollutant of the PFAS family, a worldwide issue which needs study to clarify the effects of these molecules on the development (Fenton et al., 2021). The pre-implant system resulted sensitive also to the PFOA compound, both using the gold standard MTT assay and the HCS-based technique.

We than selected TBBPA, a well-known EDC, to further study its effect on the post-implant model and investigate its mechanism of action. TBBPA is a brominated flame retardant used in a variety of consumer products, which accumulates in organisms. While producing low acute toxicity, chronic exposure may act as an ECD with serious consequences (Oral et al., 2021). Its mechanism of action at cellular level is still unclear, but involves the thyroid cellular function, interfering with the binding of thyroid hormone to its nuclear receptor beta (TRb) (Colnot et al., 2014; Stavreva et al., 2016). The flow of different *in vitro* experiments was established to reproduce the logical sequence of *in vivo* developmental toxicology testing proposed by OECD guidelines, specifically test no. 414, the Prenatal Developmental Toxicity Study (*in vivo*), which suggests administering chemicals from the preimplantation stage onwards. We then used RESCs at the pre-implantation stage for acute toxicity testing (24 h, mimicking acute exposure) to identify the TBBPA concentration required for time-course experiments (mimicking long-term exposure), with conventional viability readouts (MTT and nuclear morphology) as test results. Compared to the gold standard MTT assay, the morphology-based method was more sensitive and statistically robust. Moreover, the MTT is a colorimetric indirect method which depends on mitochondria activity, while the cell-based HCS can perform a series of parallel analysis at single cell resolution, with direct readouts quantifying cell death/viability, even using a nuclear dye only. The effects of sub-toxic doses (20, 40 µM) on cell proliferation and differentiation were then compared to vehicle alone in long-term cultures (up to 10 days) to mimic chronic toxicity. This cell system is also highly sensitive to TBBPA, although the biological effects in +/- RA are quite different (see next section). Molecular toxicology and cell lineage signatures identify both up- (*ADH1*, *CPT1B*, *NQO1*) and downregulated (*ADM2*, *SLC51A*, *SLC2A3*, *SREBF1*, *TAGLN*, *LDHA*) genes in cells exposed to 20 mM TBBPA compared to those not exposed to the chemical. In the post-implant model, acute exposure alters the expression level of 11 lineage- and 6 toxicology-related genes.

Overall, these cell systems showed high molecular sensitivity as first-line screening system. Gene expression analysis, for example, revealed that 20 µM TBBPA exposure upregulated *TBXT* mRNA expression, which is a homolog of the brachyury gene in mice, expressed on embryonic day 7.5 (E7.5) and required for notochord maintenance, mesoderm specification and axial skeleton development (Pennimpede et al., 2012). The corresponding encoded protein in humans is an embryonic T-box transcription factor affecting the transcription of genes required for mesoderm formation and differentiation (Postma et al., 2014). TBXT mutation causes congenital vertebral malformations in humans and mice (Chen et al., 2024), thus our system appears to be sufficiently sensitive to identify early genetic effects.

Compared to the gold standard mESC-based test, the RESC-based system resulted more sensitive in terms of toxicity. Moreover, the differentiation interference has been studied only in terms of selected gene expression perturbation on few selected genes (Yin et al., 2018), while we integrated a pathway analysis approach with the morphology-based high content screening data.

### 4.3 2D RESCs as a test platform to investigate the cellular mechanism of action of TBBPA

The mechanism of action of TBBPA at cellular level is still uncertain. Converging lines of evidence suggest a direct effect on the thyroid hormone nuclear receptor, as indicated by *in vivo* experiments in goldfish (Maur et al., 2022) and *in vitro* human cell systems (Koike et al., 2016). We already demonstrated that blocking the TR with a specific molecular tool acting as a pan-TR antagonist (1-850) blocks the RA-mediated neural differentiation of the RESCs (Fernández et al., 2020), prompting us to use this assay to further explore the possible mechanism of action of TBBPA. Our experiments confirmed that TR activity is necessary for the effect of TBBPA in promoting neuroectodermal differentiation; indeed, the effect of TBBPA is abolished by blocking the TR receptor via pre-exposure to the TR inhibitor 1-850. The number of nestin-expressing cells is 4–6 times higher in TBBPA-exposed cells at the pre-implant (-RA) stage, while this effect completely disappears in TBBPA-exposed cells at the post-implant (+RA) stage. Notably, TRs act as nuclear receptors on gene transcription as heterodimers, mainly with retinoid X receptors (RXRs) following ligand binding (Evans and Mangelsdorf, 2014). RESCs are a suitable model to further characterize TBBPA mode of action, as supported by our previous findings demonstrating the presence and contribution of TH and RA nuclear receptors to the process of neuroectoderm maturation (Fernández et al., 2020), in fact the role of both receptor classes in neuroectodermal differentiation, where they regulate stemness and stem cell differentiation (Jeong and Mangelsdorf, 2009), has been well-established. We then hypothesized that TBBPA may interfere with the heterodimer formation between RA receptors and TH receptors, or with one of the many and complex related regulatory molecular machineries (Lee and Privalsky, 2005). However, RA and TH pathways may contribute to the toxic profile of this compound (a phenomenon seen also when these pathways are independently activated), as occurs for other polybrominated diphenyl ethers which interfere with the TH signaling pathway by disrupting that of RA (Zhang et al., 2023). Other mechanisms, however, cannot be ruled out, including genomic and non-genomic effects. Genomic effects in particular may involve other nuclear receptors such as those for estrogen, glucocorticoids and androgens, PPARg and the aryl hydrocarbon receptor, which also varies according to species (Hung et al., 2013), while non-genomic effects include oxidative stress, intracellular organelle perturbation and altered cytokine synthesis (Yu et al., 2019). In conclusion, we believe that the proposed cellular system, based on adherent RESCs culture and potentially reducing the use of laboratory animals, may offer a useful first-line screening platform for prenatal developmental toxicity testing, being highly sensitive and based on high-throughput cellular and molecular technologies. It will, however, require full validation according to OECD guidelines prior to its proposal to the regulatory agencies. In particular, the validation pipeline will require a wide chemical library testing using standardized methods compared with the gold standard techniques, for each experimental goal, followed by a multi-centric evaluation. Besides, the full validation will necessarily include a thorough analysis of the predictive accuracy of the proposed method and a consequent comparison with the approved *in vitro* and *in vivo* methods. Regarding the test compound used in this study, TBBPA, the developmental toxicity results obtained with the proposed RESC-based method are in line with what observed in *in vivo* experiments based on rats, where the administration of this known endocrine disruptor during the gestational period showed significant behavioral effects in adults (Rock et al., 2019). Following the latest European Commission considerations (Gourmelon et al., 2024), there is an urgent need for the validation of *in vitro* methods to reduce the reliance on *in vivo* testing. The validation process must guarantee reproducibility through three key points: the in-depth description of the protocol, the data interpretation, and the admitted variability associated with the assay.

## Data Availability

The datasets presented in this study can be found in online repositories. The names of the repository/repositories and accession number(s) can be found below: https://amsacta.unibo.it/id/eprint/7847/, 10.6092/unibo/amsacta/7847.

## Abbreviations

3Rs, Reduction, Replacement, Refinement; ECVAM, European Centre for the Validation of Alternative Methods; EDC, endocrine disruptor chemicals; HCS, high content screening; IC50, inhibitory concentration 50; mEST, mouse embryonic stem cells test; OECD, organization for economic co-operation and development; RA, retinoic acid; RESC, rat embryonic stem cells; RESC-SC, Rat embryonic stem cell - single cells; ROI, region of interest; TBBPA, 2,2′,6,6′-tetrabromobisphenol A; TH, thyroid hormone; TR, thyroid hormone receptor; WEC, whole embryo culture.
