# Supplementary material for: Rat embryonic stem cell-based in vitro testing platform for mammalian embryo toxicology at pre- and post-implantation stages
Source: Front Toxicol. 2025 May 8;7:1561386. doi: 10.3389/ftox.2025.1561386 (PMC12095294; doi:10.3389/ftox.2025.1561386)
Supplement: Supplementary file 1 [file DataSheet2.pdf]

**SUPPLEMENTARY MATERIAL S4: SUPPLEMENTARY RESULTS**

A. pre-implant model (vehicle-treated): 20 μM vs 0 μM TBBPA

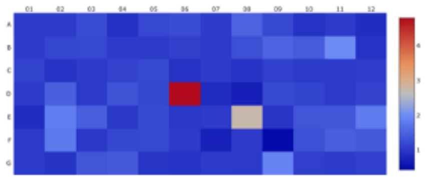

B. post-implant model (RA-treated): 20 μM vs 0 μM TBBPA

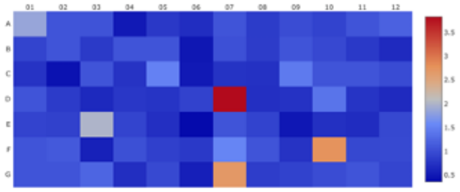

C. pre-implant model (vehicle-treated): 40 μM vs 0 μM TBBPA

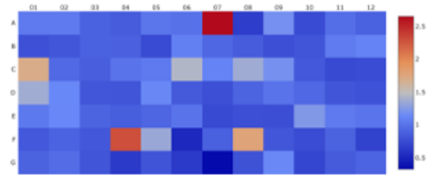

D. post-implant model (RA-treated): 40 μM vs 0 μM TBBPA

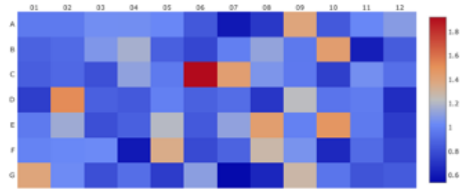

**Heatmaps of the PCRarray analysis.**

A - B) The panels show the gene expression of the entire gene set analyzed using the "Cell Lineage Identification" PCR array (PARN-508ZD, Qiagen), represented as an heatmap generated by the average fold regulation of the three replicates of TBBPA sub-toxic dose (20 μM) treated cultures compared to the control group treated with vehicle (pre-implant model, A) or RA (post-implant model, B). The heat

C – D) The panels show the gene expression of the entire gene set analyzed using the "Molecular Toxicology Pathway Finder" PCR array (PARN-401ZD, Qiagen), represented as an heatmap generated by the average fold regulation of the three replicates of TBBPA toxic dose (40 μM) treated cultures compared to the control group treated with vehicle (pre-implant model, C) or RA (post-implant model, D).
