# Supplementary material for: Rat embryonic stem cell-based in vitro testing platform for mammalian embryo toxicology at pre- and post-implantation stages
Source: Front Toxicol. 2025 May 8;7:1561386. doi: 10.3389/ftox.2025.1561386 (PMC12095294; doi:10.3389/ftox.2025.1561386)
Supplement: Supplementary file 3 [file DataSheet3.pdf]

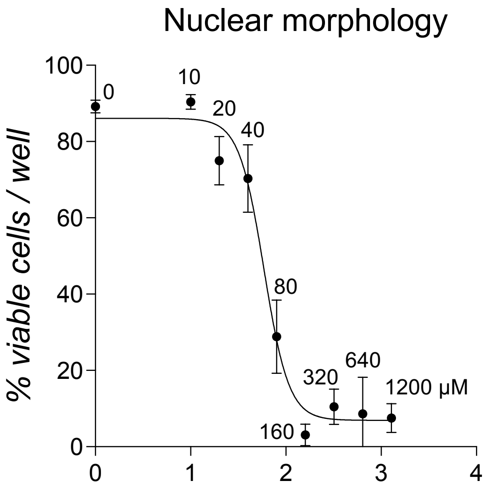

Supplementary Figure S1. HCS-based analysis of TBBPA toxicity on pre-implant model

Graph shows the cell-based HCS analysis of pre-implant cultures treated with nine different doses of TBBPA (0, 10, 20, 40, 80, 160, 320, 640, 1200 µM). Data in are expressed as percentage of viable cells per well, calculated on the total cell number per well.
