# Supplementary material for: Rat embryonic stem cell-based in vitro testing platform for mammalian embryo toxicology at pre- and post-implantation stages
Source: Front Toxicol. 2025 May 8;7:1561386. doi: 10.3389/ftox.2025.1561386 (PMC12095294; doi:10.3389/ftox.2025.1561386)
Supplement: Supplementary file 4 [file DataSheet1.pdf]

SUPPLEMENTARY MATERIAL S1: DETAILED MATERIALS AND METHODS

Cell culture medium composition

The SCML medium was composed of DMEM:F12 (Dulbecco's Modified Eagle Medium: Nutrient Mixture F-12; Gibco, Waltham, MA, USA), 10% FBS (Fetal Bovine Serum Embryonic - stem cell tested, Euroclone, Milan, Italy), 0.1 mM MEM/NEAA (Gibco), 0.1 mM Glutamax (Gibco), 0.1 µM β-mercaptoethanol, Nucleoside Mix (8 µg/mL adenosine, 7.3 µg/mL cytidine, 8.5 µg/mL guanosine, 2.4 µg/mL thymidine, 7.3 µg/mL uridine, Sigma-Aldrich, Burlington, MA, USA), 100 U/mL penicillin, and 100 µg/mL streptomycin, 4 ng/mL (Thermo Fisher Scientific, Waltham, MA, USA). Cells were seeded at a density of 5×10^4 cells/cm^2 in 96-well plates.

MTT assay full protocol

Thiazolyl Blue Tetrazolium Bromide (Sigma-Aldrich) was dissolved at a concentration of 6.5 mg/ml in PBS and diluted at a concentration of 0.5 mg/ml in OptiMem (Gibco). The culture medium was removed, and the cells were incubated for 3 h in the incubator with the MTT solution. Formazan salts were solubilized and 100 µl of the solubilization solution (80% v/v isopropyl alcohol, 10% v/v HCl 1 N, 10% v/v Triton-X100) was added to each well of a 96-well plate. The plates were then shaken for 1 h at room temperature, and absorbance was measured with a microplate reader (Bio-Rad) at 570 nm. This assay indirectly semi-quantifies cell viability, using the production of the formazan salts by active mitochondria, and is therefore biased due to the impossibility of distinguishing between dead cells and living cells with impaired mitochondria.

Immunocytochemistry and nuclear staining

Immunocytochemistry was performed to analyze the nestin expression, while nuclear staining was used to mark the nuclei and perform the cell death assay using a cell-based high-content screening (HCS) platform. To do so, cells were fixed with 4% paraformaldehyde for 30 minutes at room temperature (RT), washed three times in PBS 1× and then blocked in PBS 1× + BSA 2% for 1h at RT. The cultures were incubated with the anti-nestin antibody (1:300, Mouse, BD Pharmigen, Cat. 1173632) diluted in Triton 0.3% solution (in PBS 1×) overnight at 4°C. The plates were washed three times with PBS 1× to remove the unbound antibodies and then stained with Hoechst 33258 (Thermo Fisher Scientific) and secondary antibody anti-mouse conjugated with Alexa Fluor 488 (1:500, Invitrogen, Cat. 2428531) for 30 min at 37°C. Finally, three washes with PBS 1× were performed and the stained cultures used for image acquisition within 24 h.

RNA isolation, reverse transcription and semi-quantitative real-time PCR

The gene expression studies were performed using rt-PCR, while total RNA isolation was carried out using the RNeasy Micro Kit (Qiagen, Milan, Italy) and quantified by Nanodrop 2000 spectrophotometer (Thermo Fisher Scientific). cDNA synthesis was performed using the iScriptTMcDNA Synthesis Kit (Bio-Rad, Hercules, CA, USA) following the manufacturer's instructions, using the same quantity of RNA for all the samples. A no-reverse transcription (no-RT) sample was included in the study, containing all the reagents but RNase-free water instead of the RNA solution. Semi-quantitative real-time PCR reactions were performed in a final volume of 20 μL, using the SYBR Green qPCR master mix (Bio-Rad) following the manufacturer's instructions. Specific primers (full sequences are shown in Supplementary Table S1) were used to analyze the expression of genes involved in the pluripotency/differentiation equilibrium of the pluripotent cells. Control samples (no-RT and no-template controls) were processed for each pair of primers used. PCR reactions were performed using the following steps and thermal profile: (1) denaturation step (98°C, 3 min); (2) 40 cycles of denaturation (95°C, 10 s) and annealing/amplification (60°C, 60 s), and (3) the melting curve of the amplified products (55°C to 95°C, $\Delta t = 0.5$ ° C/s). The specificity of the PCR reactions was evinced from the single peak obtained after performing the melting curve, and from the single band of the expected size when loaded in a gel electrophoresis run. Primer efficiency values for all primers were 95–105%, therefore the $2^{(-\Delta\Delta Ct)}$ method was used to perform the analysis.

Supplementary Table S1. Gene-specific primer sequences used for RT-qPCR analysis.

Gene[1]	FW (5' – 3')	REV (5' – 3')
DKK	CTGCATGAGGCACGCTATGT	ACAGAGCCTTCTTGCCCTTTG
FOXA2	ACAAGATGCTGACGC	AATGACGGATGGAGTTCTGC
GAPDH	GGCAAGTTCAATGGCACAGTCAAG	CATACTCAGCACCAGCATCAC
KLF4	TTCTCATCTCAAGGCACACC	GTCGCACTTCTGGCACTG
NANOG	CTCCTCCGCCTTCCTCTG	GACCTTGTTCTCTTCTTTCTTCTC
OCT4	CCCGCGAGTACAACCTTCTT	CGCAGCGATATCGTCATCCA
SOX2	GGAGAAAGAAGAGGAGAGAG	CTGGCGGAGAATAGTTGG
SOX17	GCCAAAGACGAACGCAAGC	TTCTCCGCCAAGGTCAACG

[1]*DKK*, dickkopf WNT signaling pathway inhibitor; *FOXA2*, forkhead box protein A2; *GAPDH*, glyceraldehyde-3-phosphate dehydrogenase; *KLF4*, Krüppel-like Factor 4; *NANOG*, Nanog homeobox; *OCT4*, octamer-binding transcription factor 4; *SOX2*, SRY-box transcription factor 2; *SOX17*, SRY-box transcription factor 17.
